# Regional intensive care transports: a prospective analysis of distance, time and cost for road, helicopter and fixed-wing ambulances

**DOI:** 10.1186/1757-7241-22-36

**Published:** 2014-06-05

**Authors:** Helge Brändström, Ola Winsö, Lars Lindholm, Michael Haney

**Affiliations:** 1 Surgical and Perioperative Sciences, Anesthesia and Intensive Care Medicine, Umeå University, Umeå, Sweden; 2 Epidemiology and Global Health, Umeå University, Umeå, Sweden

**Keywords:** Ambulance, Intensive care transport, Helicopter, Road ambulance, Fixed-wing ambulance, Health economics

## Abstract

**Background:**

There are three different types of ambulance systems, all of which can manage the same secondary intensive care patient transport mission: road ambulance, rotor-wing ambulance, and fixed-wing ambulance. We hypothesized that costs for specific transport distances would differ between systems. We aimed to analyze distances and observed times for ambulance intensive care secondary transport missions together with system costs to assess this.

**Methods:**

We prospectively collected data for consecutive urgent intensive care transports into the regional tertiary care hospital in the northern region of Sweden. Distances and transport times were gathered, and a cost model was generated based on these together with fixed and operating costs from the three different ambulance systems. Distance-cost and time–cost estimations were then generated for each transport system.

**Results:**

Road ambulance cost relatively less for shorter distances (within 250 kilometers/155 miles) but were relatively time ineffective. The rotor-wing systems were most expensive regardless of distance; but were most time-effective up to 400–500 km (248–310 miles). Fixed-wing systems were more cost-effective for longer distance (300 km/186 miles), and time effective for transports over 500 km (310 miles).

**Conclusions:**

In summary, based on an economic model developed from observed regional ICU patient transports, and cost estimations, different ambulance system cost-distances could be compared. Distance-cost and time results show that helicopters can be effective up to moderate ICU transport distances (400–500), though are expensive to operate. For longer ICU patient transports, fixed-wing transport systems are both cost and time effective compared to helicopter-based systems.

## Introduction

In a geographically large region with centralized tertiary health care resources, intensive care transport resources are routine, utilized daily, and at all hours to optimize patient care [1,2]. Patients in need of specialized health care services and intensive care are transported as quickly as possible to their tertiary center by specialized transport teams [3]. Later, these patients are transported back by the same systems to their local hospitals when no longer in need of tertiary health care services. Intensive care transports are by definition secondary transports, since they manage patients for transport from one hospital intensive care unit (ICU) to another hospital and ICU. Even though secondary transports are often acute or time sensitive, some of these are planned or can be scheduled.

Traditionally, there are three different types of ambulance systems, all of which can manage the same secondary ICU patient transport mission: road ambulance (RA), rotor-wing ambulance (RW), and fixed-wing ambulance (FW) [4]. These ambulance systems operate in the same geographical areas, overlapping each other. There is always a need for some redundancy in the overall regional transport planning, in part to manage peak transport needs, but also to provide coverage when one or another transport system in use has operational limitations from technical or external factors, including weather restrictions and mechanical break-downs [5,6].

Devoted transport systems for ICU transports are in effect mobile intensive care units, and are extremely expensive to operate [7-9]. Once the medical equipment and personnel needs are met in an ICU transport system, both for treatment and transport timeliness, then there must be an aspect of cost management concerning means of transport [10,11]. This can be assessed on a mission-by-mission basis, or on an annual transport system budget basis. Both transport time and distance are central in determining cost for the transport system. A cost analysis would need to take into account the fixed costs for availability of the system, the operating costs for the system for that specific transport distance and time, as well as personnel costs related to that specific transport system and time. A model could then be generated for approximating costs for each type of transport system, and costs for specific distances or time in operation.

We hypothesized that, for longer distance transports, one system is more cost-effective as well as time-effective compared to the others. We aimed to test this through measuring and recording transport distances and times for each of the systems, and then comparing them. We aimed to construct an economic model for costs and times for each transport system, in order to perform this comparison for short, moderate, and long transport distances.

## Methods

### General aspects

This is a prospective analysis performed with approval of the Regional Ethical Review Board in Umeå, Sweden (diary number 2012-95-31 Ö). Predetermined and specific time points for consecutive intensive care transports by road, helicopter and fixed-wing transports were recorded by the intensive care ambulance medical personnel for the following periods: October 2008 – January 2009 for fixed-wing as part of an internal prospective review, and then June 2012 to January 2013 for road and helicopter ambulances as part of an expanded study protocol. Time points were recorded in order to measure the time intervals for the preparatory, actual transport (time with patient), and system restoration phases, and this was done in order to establish ‘templates’ for each of the individual steps in the process. For the preparatory phase, the times that were measured included the following: telephone contact from ambulance dispatch center to physician on call, time from telephone contact to decision (to go) time, time from decision until at ambulance site and mission-ready, and other preparation time. These ‘preparation’ times were added together and combined to a category which was then called pre-transport time. Times with the patient were recorded for the following events: report and handover from ICU team to transport team, transport within hospital and loading in ambulance (these times not included in the calculation since they were the same for all systems), time until pick-up by connecting local ambulance and time for local ambulance transport to local airport (both specific for fixed-wing and helicopter), time for loading from local ambulance to FW, flight time (defined as lift-off and landing times for air-ambulances) or driving time (from ambulance hall to ambulance hall), time for report and hand-over, time for loading into the ambulance, and time after completed handover, and total time with patient for medical crew. Finally, times were noted for ground transfer between FW and road ambulance without patient, back to ambulance station for cleaning/resetting/refilling in order to be mission-ready again (restoration times).

The fixed-wing aircraft employed in the air ambulance role was a two-engine turboprop with pressurized cabin (Beech 200 Super King Air), represented as FW1. The helicopter (rotor wing, RW) types employed included the following: Eurocopter AS 365 N3 Dauphin (RW1) and Eurocopter AS 365 N2 Dauphin (RW2 and RW3), which both have the same cruising speed, though different lift capacities. The transit travel speed (template) for the FW was 500 km/hr (310 miles/hr), for RW was 270 km/hr (167 miles/hr), and for road ambulance (RA) 70 km/hr (42 miles/hr). The local RA speed for moving FW-transported patients from the airfield to the receiving hospital was set at 50 km/hr (31 miles/hr).

Cost estimations for each of the ambulance systems were based on information provided by the hospital administrator responsible for the regional ambulance contracts with the private ambulance vendor, and were based on the ambulance contract itself, and are provided in Swedish kronor (SEK). The costs for medical crews are the costs reported by each of the county councils for the medical crew cost (for year 2012). Annual flying hours for the FW and RW systems were based on the averages from the previous two years. For the RW1, measured for the Norrbotten county, Gällivare city system with two pilots as aircrew, the total operating costs per hour (hr) were derived from an estimated annual activity of 750 flying hours, fixed costs of 37,000 SEK/hr, operating costs of 12,800 SEK/hr, personnel costs of 11,000 SEK/hr (one specialist physician and one specialist nurse as medical crew), administrative costs of 900 SEK/hr. This lead to a total hourly mission cost of 61700 SEK/hr.

For the RW2, measured for the Västerbotten county, Lycksele city system with two pilots as aircrew, and for an estimated annual activity of 700 flying hours, fixed costs of 25,714 SEK/hr, hourly operating costs of 8,000 SEK/hr, personnel costs 7,895 SEK/hr (one specialist physician and one specialist nurse as medical crew), administrative hourly 71 SEK/hr, this allowed us to derive an hourly mission cost of 41,610 SEK/hr.

For the RW3, measured for the Jämtland county, Östersund city system, and for an estimated annual activity of 737 flying hours with one pilot and one pilot assistant (helicopter emergency medical service crew/HEMS) for aircrew, fixed costs of 24,483 SEK/hr, hourly operating costs of 9,500 SEK/hr, personnel costs 3,053 SEK/hr (one specialist nurse alone as medical crew), administrative hourly 814 SEK/hr, this allowed us to derive an hourly mission cost of 37,850 SEK/hr.

For the FW system, measured for the Northern Sweden region, operating with two pilots and a specialist nurse for aircrew, and for an estimated annual activity of 2,400 flying hours (based on previous 2 years activity), fixed costs of 0 SEK/hr, hourly operating costs of 21,301 SEK/hr, personnel costs 4,430 SEK/hr (specialist physician and specialist nurse as medical crew), administrative hourly 100 SEK/hr, this allowed us to derive an hourly mission cost of 25,831 SEK/hr.

For the RA system, the estimation was based on a per-kilometer cost, from the county council’s own estimation of costs based on approximately (nationally) 8,200,000 SEK per ambulance per year, and 100,000 kilometers, leading to an estimation of 82 SEK per km. This was converted to an hourly cost of 5,740 SEK/hr, based on average transit speed of 70 km/hr (43 miles/hr). For the RA system, there was typically an additional nurse from the ‘sending’ hospital accompanying the patient. These cost estimations were simplified to the following, to ease further calculations: RW1 60,000 SEK/hr, RW2 and RW3 40,000 SEK/hr, FW 25,000 SEK/hr, and och RA 6,000 SEK/hr.

Cost-distance is defined as the cost per km patient transportation with the given ambulance system. This was calculated from the cost per hour of operation for each ambulance system using the template for travel speed during transit for each of the systems: RA = 70 km/hr, RW 270 km/hr and FW 500 km/hr. For FW, the road ambulance connections between airport and hospital (template distance 10 km, 50 km/hr, medical crew plus RA costs for this segment which add up to 9,970 SEK for both sending and receiving hospital/airport connections) were included in the time and speed assessment.

Cost estimates for each ambulance system are based on reports from each of the county councils (Table 1), and consist of ‘costs’ in the contracts with the private air ambulance companies for operational support for public hospital missions, as well as different county council contracts and medical crew salaries. The FW fixed costs are not presented separately in the contracts, but are rolled into hourly costs, and these are presented. Only the helicopter systems here present fixed costs. These different RW systems include one (with HEMS) and two pilot (no HEMS) helicopter aircrew configurations. Medical staffing in the ambulance is shown for each system (Table 1), and was either 2 nurses, or a physician and nurse. Physician and nurse costs are presented as an hourly mission cost, and these were collected for each county and ambulance system. Medical crew costs included ‘on-call’ availability costs.

**Table 1:** Ambulance system cost estimations

**System**	**Total**	**Fixed cost**	**%**	**Op cost (fuel, crew)**	**Hourly%**	**Total cost, excl med crew**	**%**	**Med crew**	**Med crew%**	**Admin**	**Admin%**
RA	6,040 SEK/hr	included		included		82, SEK/km 57,740 SEK/hr	95%	300 SEK/hr	5%	included	
RW 1	61,700 SEK/hr	37,000 SEK/hr	60%	12,800 SEK/hr	21%	50,700 SEK/hr	82%	11,000 SEK/hr	18%	900 SEK/hr	1%
RW 2	41,610 SEK/hr	25,714 SEK/hr	62%	8,000 SEK/hr	19%	33,715 SEK/hr	81%	7,895 SEK/hr	19%	71 SEK/hr	< 1%
RW 3	37,850 SEK/hr	24,483 SEK/hr	65%	9,500 SEK/hr	25%	34,947 SEK/hr	92%	3,053 SEK/hr	8%	814 SEK/hr	< 1%
FW	25,831 SEK/hr	none in this contract		21,301 SEK/hr	100%	21,301 SEK/hr	82%	4,430 SEK/hr	18%	100 SEK/hr	< 1%

Table legend. RA = road ambulance, RW = rotor wing ambulance, FW = fixed wing ambulance. For RA: usual ambulance crew plus addition specialist nurse as medical crew; Op = operational; hr = hour; excl = excluding; Med = medical; Admin = administration cost; For RW1 and RW2: two pilot and physician plus nurse medical crew. For RW3: one pilot plus HEMS pilot assistant and nurse only medical crew. For FW: two pilot and physician plus nurse medical crew.

In addition to the distance-cost estimation (based on the above schematized average speeds for each system), there was added a schematized start-cost based on time on the ground with patient. For all the RW systems, the time in the helicopter before and after flying was estimated to be 4 minutes total (aircraft start-up and shut-down): RW-1 4,000 SEK, RW-2 and RW-3 2,667 SEK. For the FW system, the patient time in the aircraft before flying was estimated to be 10 minutes before take-off and 10 minutes after landing, for a total of 20 nonflying minutes in the aircraft, and this was added to the 12 minutes (times 2) for each ground ambulance transport from or to hospital. This resulted in 9,970 SEK standing ground costs for each FW transport mission. The RA system reported a start-up cost of 1,640 SEK, which was the standing start cost for all RW transport missions.

### Analysis

The primary endpoint was demonstration of total cost for ICU transport distance for the different transport systems, with focus on moderate and longer transport distance ranges. A secondary endpoint was to analyze the relation between distance and transport time for the different systems. Linear regression and calculation of a correlation coefficient was performed for distance-time relations. Testing for differences between categories and groups for measured values was performed using a Mann-Whitney U test. Significant differences in comparisons were noted when there was a p value <0.05.

## Results

### Transport times (observed) and distance

**Figure 1:**
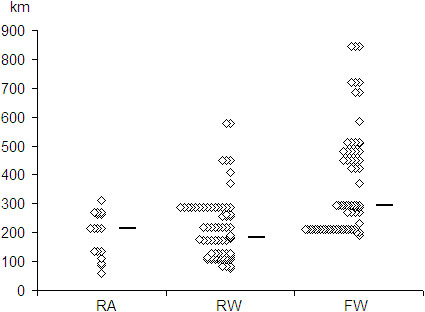
**Transport distances with patient, different transport systems.** These observed transport distances (with median marked), for each system, are the basis for calculation of transport costs. For obvious logistical reasons, the longest transport distances are managed by the fixed-wing system, even though there is quite a bit of overlap for moderate transport distances (200–400 km) RA = road ambulance (n = 17); RW = rotor wing ambulance (n = 71); FW = fixed-wing ambulance (n = 68).

**Figure 2:**
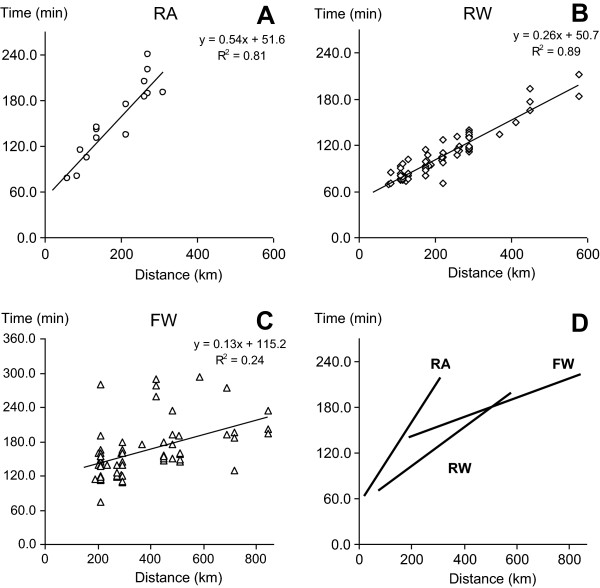
**Transport times and distance with patient.** Panels **A**, **B**, and **C** show observed transport times plotted versus distance for each of the transport ambulance systems: RA = road ambulance (n = 17), RW = rotor wing ambulance (n = 71), FW = fixed-wing ambulance (n = 68). Panel **D** shows the linear regression lines (with regression equations and R2 values in each panel, **A**, **B C**), with the observed minimal and maximal time and distance limits. Note in that even before 200 km transport distance, RA is slower than the others, even including transport preparation time. At transport distances above approximately 500 km, estimated times for transport with FW become shorter than those with the other systems.

For the RA system (n = 17), transport mission times and distances were included for analysis: 143 minutes, 77–240; 213 kilometers (132 miles), 60–310 respectively (median and range values). For the RW system (n = 71), transport mission times and distances were included for analysis: median 100 minutes with range 69–212; median 184 kilometers (114 miles) with range 76–578 (47–359 miles). For the FW system (n = 68), transport mission times and distances were included for analysis: median 155 minutes with range 75–293 and 293 kilometers (182 miles), range 190–844 kilometers (118–524 miles). The number of transports as specific distances by system is shown in Figure 1. The observed domestic transports with FW did not exceed 900 km (559 miles). The times-distance relationships are presented for each system in Figure 2.

Time with patient in relation to distance results was analyzed by linear regression. For the RA system, y = 0.54× + 51.6 (R^2^ = 0.81), for the RW system, y = 0.26× + 50.7 (R^2^ = 0.89), and for the FW system, y = 0.13× + 115.2 (R^2^ = 0.24), where y = time (minutes) and x = distance (kilometer). Note that the strength of the correlation between time and distance was weak for the FW group.

Another aspect of the ambulance missions was ambulance time and distance without patient, where the ambulances either needed to travel to get to the patient, or where the ambulance needed to travel after the patient transport, in order to get back to the ambulance station. Concerning percentage of total mission time with patient, all 3 systems carried patients approximately half of their transit time (base on ration of mean values): RA 44%, RW 50%, and FW 49%, and no difference was detected between these groups.

### Costs, ambulance systems and distances

**Figure 3:**
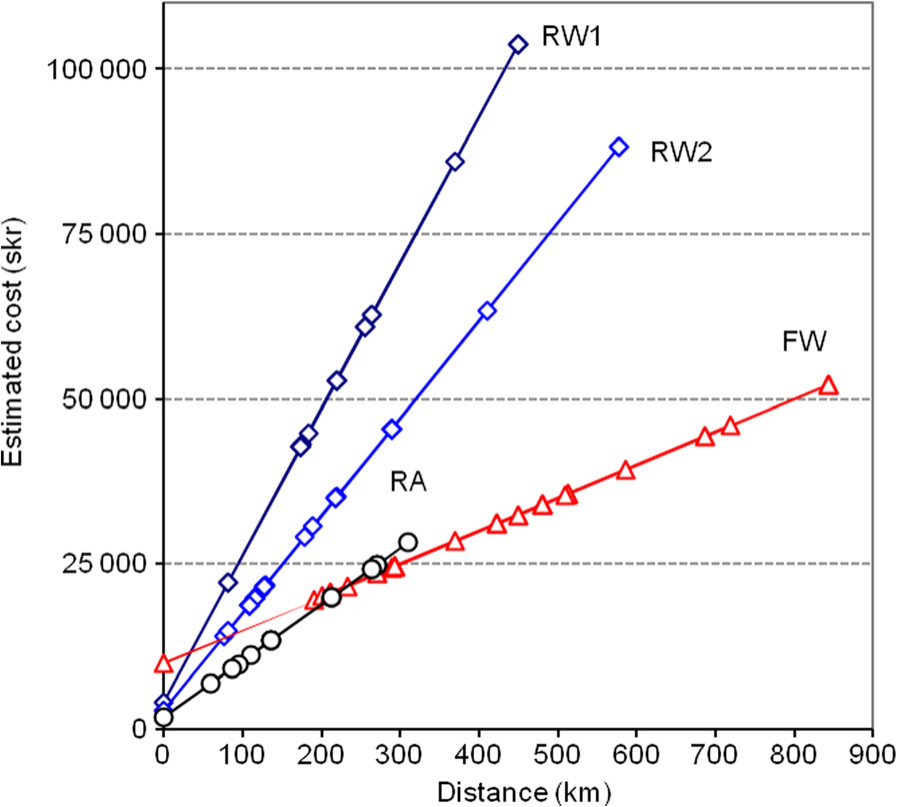
**Transport distances with patient, and estimated costs by system and distance.** RA = road ambulance (n = 17); RW = rotor wing ambulance (n = 71); FW = fixed-wing ambulance (n = 68). Individual points on each line represent transport distances where there can have been many actual transports superimposed. Helicopter transport costs by distance and patient time are clearly more expensive than RA and FW systems. Different helicopter models and crew configurations lead to different operating costs over distance and transport mission, though RW costs are always more than double that of the other systems related to distance, though the relative time-effectiveness of helicopter missions reduces relative mission time for shorter transports (below 500 km), as shown in Figure 2. Since there were multiple transports over the same distances (between two hospitals), only the unique distances are noted on the regression lines. RW1 represents Eurocopter AS 365 N3 Dauphin, and RW2 represents Eurocopter AS 365 N2 Dauphin configurations.

From the cost estimations from each of the County Councils their ambulance systems, estimated costs per operating minute are presented in relation to distances. Observed distances together with schematized costs (Figure 3) demonstrate that RA is least expensive up to 250 km transport distance, that over 250 km FW is least costly, and that RW transports are the most costly regardless of distance. Helicopter ambulance missions did not exceed 800 km in distance, and road ambulance intensive care missions did not exceed 700 km.

## Discussion

Based on actual observed missions, we were able to generate a model for distance-costs and time costs for each ambulance system that managed ICU transports. Using this model of cost-estimations for activities, comparisons were demonstrated for the ICU transport system costs. This analysis demonstrates that for moderate transport distances (150–250 km), the RA and FW transport systems performed similarly in terms of time and cost, though with diverging transport times and costs above 250 km (RA slower and more expensive). The RW system is the most expensive ICU patient transport system, regardless of distance, though it is still often employed, presumably based on availability and transport timeliness. These observations agree with previous reports, where a similar range of RW ambulance costs have been described, along with medical effectiveness for urgent critical care transport [7-11]. This report is unique in presenting RW, FW, and RA ICU transports together from the same coordinated regional system.

The FW system was consistently employed for long (more than 250 km) or very long (more than 600 km or 372 miles) transports, where these results also show a clear cost and time advantage compared to the other systems. The FW system becomes more cost effective, compared to RA, at approximately 250 km transport distance, and these differences increase with increasing distance. It is important to note that all FW transports employ local RA resources between hospital and airport (these costs included in the FW estimates), in contrast to most RW systems which do not require this local support.There are similar transport times for all three systems when there were shorter or moderate distance transports. For longer transports (over 500 km), the higher velocity of the FW transport is associated with shorter expected transport times (Figure 2, panel D). All three transport systems have a ‘built-in’ preparation time, where the medical teams are managing the patient, but the ambulance is not yet moving, and these results show that there are similar preparation times for all three systems (includes patient reporting, ground transport before entering the ambulance, loading and safety checking before ambulance start.

We observed cost differences between the three different RW ambulance systems examined here. We interpret this as a sign that there can be quite a bit of variation in the operating costs for RW helicopter ambulance systems. In this analysis, we included only the contractual expenses to the health authority for aircraft and aircrew services, and did not have access to the private air ambulance company’s actual operating costs. All three systems employed a similar medium-weight multi-purpose twin-engine helicopter, though with variation in motor lift capacity, pilot configuration, and medical crew configuration. These details clearly contribute to RW ambulance system cost differences.

Concerning costs, risks, and optimal use of transport resources, there are several considerations. When choosing an optimal ambulance system for a particular secondary ICU transport mission, this will have implications as far as time without that resource locally, or for other acute secondary transport missions which might arise. These different ambulance systems always interact regionally, and to some extent overlap in their capabilities, and are dependent upon each other. When one ambulance resource is employed in a secondary (ICU) transport mission, then it is not available for other local or regional ambulance missions. When one system is occupied, then the other systems may need to become involved in acute secondary ICU transport missions over distance, even if their main mission is local primary ambulance retrieval. Therefore, there is no practical experience with recruiting more physician and nurse personnel on short notice due to heavy ambulance ICU transport activity, and we have not tried to estimate these theoretical ‘opportunity’ costs. Regional coordination is clearly needed, in order to meet both local primary and regional secondary transport needs with these overlapping ambulance resources. Also, each of the 3 system types is absolutely necessary, since the patient transport missions have such a wide range of needs (time, distance, medical acuity). If transport missions take less time, then the medical personnel and ambulance vehicles are available more of the time. This would be very important if there was a very heavy burden of intensive care transports each day, but this was not the case in the observed region, where in the whole region there were on average between one and two intensive care transport missions per day. Additionally, one can consider the risks related to ambulance transport, where RW and RA systems have higher risk for accident and are more weather-dependent. These considerations for safety are important. For longer transports, FW systems are considered safer and preferred in order to avoid the inherent risk of helicopter or road transport.

The need for coordination is most clearly demonstrated by the observation from each of the systems that they consistently travel empty (without patient) on the way to, or on the way from ICU patient transports. For optimal use of this expensive ambulance resource, even non-urgent and non-ICU patients waiting for transport for the same routes might be served, though this may not always be practical. There are other patient transport systems (large road ambulance) and planned non-ICU FW operating on a scheduled basis, and successful coordination and integration with urgent or emergent ICU missions was not observed. In the model generated here, since one leg of the ICU transport mission was almost always traveled empty (without patient), then that can possibly be regarded as part of the ICU transport cost.

For FW transports, there are always two RA local transports involved, though these exact times and distances were simplified in this material to a general average for all cities and airports. Coordination of the different logistical steps is important in making these secondary FW missions time- and cost-effective. If there is a problem with local RA availability before FW start or after FW landing at an airport, then this will significantly prolong the transport time and increase the FW transport cost, (which will be standing still). At times, all three systems can contribute in stages to transport of a single patient, where none of the 3 had sufficed by itself. Clearly, in order to accomplish acute secondary ICU patient transports in all conditions, overlapping RA, RW and FW systems are needed, together with careful coordination of missions.

Transport times for the different systems were assessed only from patient hospital bedside at the hospital which was sending. For actual transport times, the RW system took the least time transport over distances up to approximately 400 km, where then FW was faster. However, this time measurement reflects only the actual transport time (plus a schematized alarm and mobilization time), that is, the start point (sending hospital) was made the same for all systems. There was no attempt to include in the analysis the pre-transport location or status of the different ambulance systems. All hospitals in the study region (12), plus several outside of the region sending or receiving study region patients, had own RA resources. Most, however, had no RW or FW resources close by at the time of when the transport was activated. Therefore, in order to examine the patient transport specifically, the starting point was standardized to the patient location at the start of the transport. Therefore, the analysis of the time aspect of transport is limited in this respect. In the setting of acute or time-sensitive intensive care transports, the location of the air ambulance at the time of the alarm is crucial, since air ambulance alternatives can be quite a distance from sending hospital at the time of the alarm. This means that while both RW and FW have faster travel speeds, there can be cases where (despite limited speeds of RA in transit) the total time from alarm to intensive care patient delivered to receiving hospital can be shorter with RA even up to 300 km or longer.

In this material, only secondary intensive care transport activity was analyzed. There were relatively few long distance RA intensive care transport observations in these results, and this was because they were infrequent. The largest amount of activity for RA and RW ambulance systems involves primary emergency patient retrieval missions. Rotor wing and RA emergency medical systems are funded and established first and foremost for local or regional primary emergency ambulance missions, or in other words, collecting patients from sites of injury or illness, and taking them as fast as possible to the first receiving hospital. When available, RW and RA systems can be used for secondary inter-hospital ICU transport missions.

The largest amount of activity for FW systems involves non-intensive care, non-time sensitive missions (from a medical perspective). Yet, as with hospital planning for critical care services, these systems must be designed to be robust for all predictable acute patient missions. If they are designed with known performance limits that do not match needs, then the medical results will be suboptimal and cost-effectiveness will be limited, and there can be negative patient consequences. On the other hand, planners need to avoid ‘unnecessary’ costs or duplications in ICU transport systems. Some degree of overlap is needed in order to always ensure adequate ICU patient transport in all conditions. It may be difficult to identify portions of this overlapping which are not effective or not necessary. One main issue here is how to use the RW resource optimally in a secondary ICU transport role, given its high fixed- and operating costs, when dedicated long distance (FW) ICU transport systems are also available, not nearly as timely for the same missions. Since medical indications and air ambulance proximity to a patient determine which system is chosen, this then becomes a policy question of how much of each type of transport system to establish and maintain, and where to position these in order to optimize patient access. Healthcare policy makers will recognize the need for multiple overlapping systems for regional and national intensive care patient transport, though they will also recognize the need for rigorous regional (and national) coordination so that appropriate ambulance investments and operational resources are established maintained.

## Conclusions

In summary, based on an economic model developed from observed regional ICU patient transports, and cost estimations, different ambulance system distance-cost could be compared. Road ambulance systems are cost-effective for shorter distances (within 250 kilometers) but are patient-time ineffective already at shorter distances. The RW systems are always most expensive, regardless of distance; but are most transport time-effective up to 400–500 km. Fixed-wing ICU transport systems are more cost-effective for longer distance (300 km), and patient-time effective for transports over 500 km.

## Competing interests

The authors declare that they have no competing interests.

## Authors’ contributions

HB has participated in study design, data collection, data analysis and manuscript writing. OW has participated in study design, data analysis and critical review of the manuscript. LL has participated in study design, data analysis and critical review of the manuscript. MH has participated in study design, data collection, data analysis and manuscript writing. All authors have approved the final manuscript.

## Authors’ information

Helge Brändström is the section head for air ambulance services at the Intensive Care Medicine and Post-Operative Medicine Department, University Hospital of Umeå, Umeå, Sweden. Helge Brändström is also the Medical Director for the Swedish National Air Medevac system, Swedish Civil Contingencies Agency and medical advisor on aeromedical evacuation for the Swedish National Board of Health and Welfare.
